# Exercise as a Multisystem Adjunct for Alcohol-Associated Liver Disease: Inflammatory Mechanisms, Muscle–Liver Crosstalk, and Translational Gaps

**DOI:** 10.3390/metabo16090666

**Published:** 2026-09-10

**Authors:** Jing Xu, Kai Sang, Junjun Zhang

**Affiliations:** 1 Department of Police Tactics, Fujian Police College, Fuzhou 350007, China; 2 Division of Sports Science and Physical Education, Tsinghua University, Beijing 100080, China

**Keywords:** alcohol-associated liver disease, exercise, inflammation, gut–liver axis, sarcopenia

## Abstract

**Highlights:**

**What are the main findings?**

- This structured narrative review distinguishes physical activity associations from evidence on structured exercise training in ALD.
- Ethanol exposure connects redox stress, lipid intermediates, bile acids, acylcarnitines, microbial metabolites, and muscle–liver signaling.
- Most proposed exercise mechanisms remain hypothesis-generating because ALD-specific human intervention trials are scarce.

**What are the implications of the main findings?**

- Exercise should be considered stage-specific adjunctive support, not a stand-alone therapy for ALD.
- Future trials should integrate metabolomics, inflammatory biomarkers, functional outcomes, safety endpoints, AUD status, sex, and ALDH2 genotype.

**Abstract:**

**Background:** Alcohol-associated liver disease (ALD) involves ethanol-related metabolic injury, inflammation, gut–liver dysfunction, and progressive loss of skeletal muscle and functional reserve. Exercise could influence several of these pathways, but ALD-specific evidence remains limited. We therefore evaluated exercise as an adjunct, not a substitute for established care, across the ALD spectrum. **Methods:** We conducted a structured narrative review of PubMed/MEDLINE, Google Scholar, and publisher records updated through 20 August 2026. Evidence was classified as direct human ALD/MetALD evidence, direct preclinical alcohol-injury evidence, indirect clinical evidence from MASLD or mixed-etiology cirrhosis, or mechanistic evidence from broader exercise biology. **Results:** Direct human evidence is predominantly observational. Leisure-time physical activity is associated with lower liver mortality across drinking patterns and with lower odds of at-risk advanced fibrosis in MetALD/ALD. These associations do not establish the efficacy of structured exercise training. Preclinical alcohol-injury models support redox- and Nrf2-mediated hepatoprotection, whereas proposed effects on AMPK-SIRT1-PGC-1α signaling, mitochondrial quality, gut microbial metabolites, myokines, and ammonia handling remain largely extrapolated. Metabolomic studies identify disturbances in redox balance, lipid intermediates, acylcarnitines, bile acids, and amino acid and tryptophan metabolism. These alterations provide candidate endpoints for future trials. Myostatin and decorin are associated with ALD severity, but their responsiveness to exercise is unknown. We therefore propose a stage-specific research and practice framework with explicit safety considerations. **Conclusions:** Exercise is a promising but unproven multisystem adjunct for ALD. It should complement abstinence support, alcohol use disorder care, nutrition, and standard medical management. ALD-specific trials should integrate metabolomics, liver outcomes, functional measures, sex, alcohol exposure, disease stage, and adverse events.

## 1. Introduction

Alcohol-associated liver disease (ALD) is a leading cause of advanced liver disease and liver transplantation worldwide [1,2,3,4,5,6]. Its clinical spectrum extends from alcohol-associated steatosis and steatohepatitis to severe alcohol-associated hepatitis, progressive fibrosis, cirrhosis, hepatocellular carcinoma, and transplant candidacy [1,2,3,7]. Many patients first present after portal hypertension, decompensation, or severe hepatitis has developed [1,2,3,7].

The terminology for steatotic liver disease has also evolved. A multisociety Delphi consensus established steatotic liver disease as an umbrella term and defined MASLD and MetALD as clinically relevant categories [4]. MetALD denotes hepatic steatosis with metabolic dysfunction and alcohol intake above MASLD thresholds but below levels typically used to define primarily alcohol-associated disease. These ranges are commonly 140–350 g/week for women and 210–420 g/week for men [4]. Evidence from MASLD exercise studies may inform shared metabolic pathways. However, it should remain hypothesis-generating for ALD unless ALD or MetALD populations are studied directly.

Exercise is relevant to ALD because it affects multiple organ systems. Structured exercise can modify skeletal muscle substrate use, insulin sensitivity, mitochondrial remodeling, adipose inflammation, gut microbial ecology, immune tone, endothelial function, and functional reserve [8,9,10,11,12,13,14,15,16]. Several of these processes are disrupted by alcohol exposure. However, patients with ALD may have conditions that limit the applicability of general exercise recommendations. These include active alcohol use, withdrawal risk, malnutrition, anemia, cardiomyopathy, peripheral neuropathy, hepatic encephalopathy, ascites, infection, and frailty [17,18,19].

This review does not present exercise as an established treatment for ALD. Instead, it provides a structured narrative and critical translational synthesis. First, we distinguish observational associations with physical activity from evidence on structured exercise training. Second, we separate direct ALD/MetALD evidence from indirect evidence in MASLD, mixed-etiology cirrhosis, and general exercise biology. Third, we organize the literature around metabolite-level and interorgan mechanisms relevant to *Metabolites*. Finally, we propose stage-specific research priorities rather than definitive exercise prescriptions. This cautious framing reflects the scarcity of disease-specific randomized exercise trials in ALD.

## 2. Materials and Methods

We conducted a structured narrative review rather than a systematic review, scoping review, or meta-analysis. Accordingly, we did not undertake duplicate screening, formal risk-of-bias assessment, PRISMA flow documentation, or quantitative pooling. The search was updated through 20 August 2026 and covered PubMed/MEDLINE, Google Scholar, and publisher records used to verify citations. Search terms included alcohol-associated liver disease, alcohol-related liver disease, alcoholic hepatitis, MetALD, MASLD, steatotic liver disease, exercise, physical activity, resistance training, aerobic training, cirrhosis, frailty, sarcopenia, myokines, myostatin, decorin, gut–liver axis, microbiota, metabolomics, acylcarnitines, bile acids, tryptophan, AhR, ALDH2, Nrf2, AMPK, PGC-1α, and inflammasome.

Eligible sources included clinical guidelines, nomenclature statements, major reviews, mechanistic studies of ALD pathogenesis, and preclinical alcohol-injury exercise models. We also included observational ALD or MetALD studies, exercise trials or syntheses in MASLD and mixed-etiology cirrhosis, and studies of metabolomics, gut-derived metabolites, myokines, sarcopenia, or frailty. We excluded duplicate or superseded records, articles unrelated to alcohol-associated or steatotic liver disease, and reports without mechanistic or translational relevance to exercise. When several papers supported the same point, we prioritized recent guidelines, original ALD/MetALD studies, and studies with clearly defined outcomes.

We classified evidence into four levels. Level 1 comprises direct human ALD/MetALD evidence, which is currently mainly observational. Level 2 comprises direct preclinical alcohol-injury evidence. Level 3 includes indirect clinical evidence from MASLD, MetALD-related metabolic pathways, or mixed-etiology cirrhosis rehabilitation. Level 4 represents mechanistic plausibility derived from general exercise biology or non-ALD liver disease. This hierarchy is applied throughout the manuscript and summarized in Supplementary Table S1. Its purpose is to prevent extrapolated findings from being presented as established ALD therapy.

## 3. Clinical Stages and Terminology

We consistently use the term alcohol-associated liver disease, except when citing titles or guidelines that use alcohol-related liver disease or alcoholic liver disease. ALD encompasses several clinically distinct states. Alcohol-associated steatosis primarily reflects hepatic fat accumulation and may resolve with abstinence. Alcohol-associated steatohepatitis adds hepatocellular injury and inflammation. Severe alcohol-associated hepatitis is an acute syndrome characterized by jaundice and high short-term mortality. Compensated cirrhosis differs fundamentally from decompensated cirrhosis, in which ascites, variceal bleeding, encephalopathy, infection, or renal dysfunction may affect exercise safety. Transplant candidates have additional prehabilitation needs. Active alcohol use or AUD may also affect adherence, nutrition, injury risk, and relapse outcomes [1,2,3,7,18,19].

MASLD and MetALD should be distinguished from ALD. MASLD requires hepatic steatosis plus at least one cardiometabolic risk factor, including excess adiposity, impaired glucose regulation or diabetes, hypertension, hypertriglyceridemia, or low HDL cholesterol [4,20]. MetALD combines MASLD-type metabolic dysfunction with alcohol intake above low-risk thresholds [4,20]. Exercise findings in MASLD may inform shared metabolic pathways. However, they cannot be assumed to apply to ALD dominated by acetaldehyde toxicity, active AUD, malnutrition, immune activation, or decompensation. Supplementary Table S2 summarizes the stage-specific terminology and translational approach used in this review.

**Figure 1:**
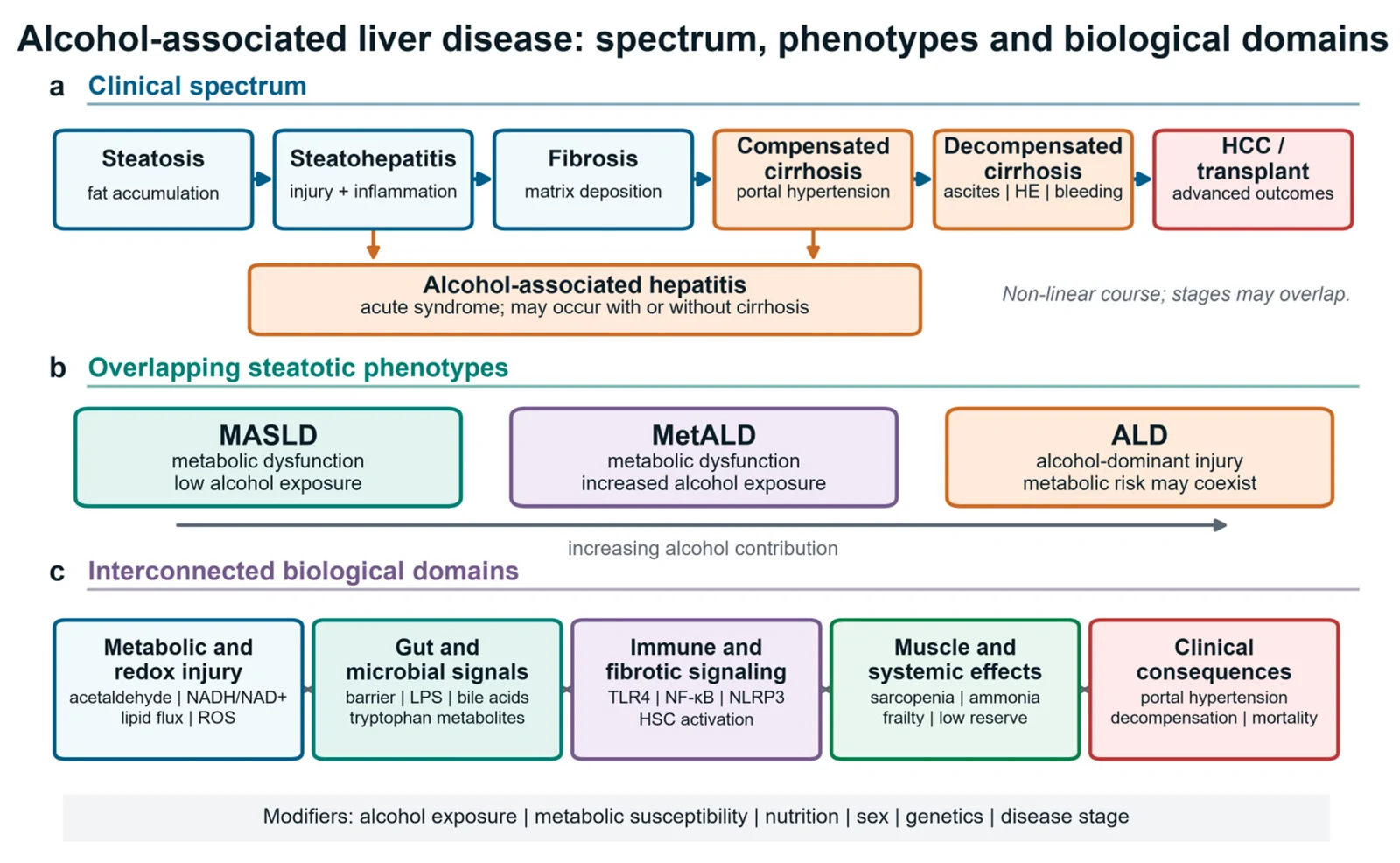
(**a**) The non-linear clinical spectrum extends from steatosis to advanced outcomes; alcohol-associated hepatitis may occur with or without cirrhosis. (**b**) MASLD, MetALD, and ALD are positioned along a continuum of increasing alcohol contribution, while metabolic dysfunction may coexist across phenotypes. (**c**) Metabolic and redox injury, gut and microbial signals, immune and fibrotic signaling, muscle and systemic effects, and clinical consequences interact bidirectionally. Alcohol exposure, metabolic susceptibility, nutrition, sex, genetics, and disease stage modify these domains.

Figure 1 shows the clinical spectrum, overlapping steatotic phenotypes, and interconnected biological domains of alcohol-associated liver disease (ALD).

## 4. Metabolic and Inflammatory Mechanisms of ALD

ALD begins with hepatic ethanol metabolism. Ethanol oxidation increases acetaldehyde exposure, shifts the NADH/NAD+ ratio, suppresses fatty acid oxidation, promotes lipogenesis, and impairs mitochondrial function. CYP2E1 induction further increases ROS generation and lipid peroxidation [21,22,23,24,25,26]. These changes help explain the potential biological relevance of exercise. Exercise alters substrate flux, mitochondrial remodeling, and redox adaptation, but its interaction with ethanol-driven metabolic injury remains inadequately tested in human ALD.

A metabolite-centered perspective also aligns this review with the scope of *Metabolites*. Serum metabolomic profiling in acute alcohol-associated hepatitis has revealed broad disturbances in amino acid, lipid, bile acid, and energy-related pathways [27]. Serum acylcarnitines are associated with high short-term mortality in alcohol-associated hepatitis, consistent with impaired mitochondrial fatty acid handling and incomplete β-oxidation in severe disease [28]. More recent proteomic and metabolomic studies have characterized MetALD and separated metabolic from alcohol-associated contributions to steatotic liver disease [29]. These findings support metabolomic endpoints in future exercise trials. Candidate measures include acylcarnitines, bile acids, aromatic amino acids, tryptophan metabolites, lactate/pyruvate-related redox markers, lipid intermediates, and gut-derived metabolites.

Inflammatory amplification drives progression from metabolic stress to tissue injury. Ethanol-induced barrier dysfunction increases portal exposure to LPS and other microbial products. These signals activate Kupffer cells and recruited macrophages through TLR4, NF-κB, inflammasome, and cytokine networks [30,31,32,33,34,35,36]. IL-1 signaling is experimentally relevant because IL-1 receptor antagonism ameliorated inflammasome-dependent alcoholic steatohepatitis in mice [30]. Persistent inflammation promotes HSC activation, extracellular matrix deposition, sinusoidal remodeling, and portal hypertension, thereby linking fibrogenesis to clinical outcomes [37,38,39].

Exercise may influence upstream drivers of this network, including insulin resistance, visceral adiposity, mitochondrial quality control, oxidative stress, and systemic inflammation [8,9,10,11,12,13,20,40,41,42,43,44,45,46]. These relationships should be considered candidate pathways rather than established ALD mechanisms. To date, no human ALD intervention study has shown that exercise improves liver-specific outcomes by activating hepatic AMPK-SIRT1-PGC-1α, Nrf2, gut microbial metabolite, or inflammasome pathways.

## 5. Gut–Liver Axis and ALDH2

The gut–liver axis is central to alcohol-associated inflammation. Alcohol disrupts epithelial barrier integrity, alters microbial ecology and bile acid signaling, and increases the portal delivery of bacterial products [32,33,34,35]. Among people with alcohol dependence, increased intestinal permeability and gut dysbiosis are associated with behavioral markers of disease severity [34]. In severe alcohol-associated hepatitis, cytolysin-positive Enterococcus faecalis has been identified as a disease-modifying organism. Targeted bacteriophage treatment attenuated alcohol-associated liver disease in experimental models [35].

Microbial metabolites provide another potential link. In human microbiota-associated mouse models, microbiota-derived tryptophan metabolites activated AhR and reduced alcohol-induced liver injury [47]. This finding does not show that exercise improves AhR signaling in human ALD, but it defines a testable metabolite-level pathway. Studies outside ALD indicate that exercise can alter gut microbial composition and function [14,15]. Microbiome outcomes in ALD trials require cautious interpretation. Diet, alcohol consumption, antibiotics, proton pump inhibitors, obesity, disease severity, hospitalization, and cirrhosis complications may all confound microbial and metabolite measurements.

ALDH2 illustrates how genetic susceptibility may modify alcohol-related injury. In mice, ALDH2 deficiency increases susceptibility to binge alcohol-induced gut leakiness, endotoxemia, oxidative and nitrative stress, and acute liver injury through the gut–liver axis [48]. Human genetic studies also support its relevance. Among middle-aged Japanese men, ALDH2 genotype modified the association between alcohol consumption and the AST/ALT ratio [49]. These observations are particularly relevant to East Asian populations. However, the effects of ALDH2 genotype or experimental ALDH2 deficiency on exercise responses in human ALD remain unknown.

## 6. Human Evidence for Physical Activity and Exercise

Physical activity and exercise are distinct concepts. Physical activity includes any bodily movement that increases energy expenditure, including occupational, household, transport, and leisure-time activities. Exercise is a planned, structured, and repetitive form of physical activity intended to improve or maintain fitness. Associations with physical activity do not establish the therapeutic efficacy of prescribed exercise training. This distinction is especially important in ALD because occupational activity may reflect socioeconomic adversity, long working hours, insufficient recovery, or physically demanding labor rather than adaptive training.

A US population study linked 60,334 adults to mortality follow-up through 31 December 2019 and recorded 252 liver-specific deaths over a median of 12.2 years [50]. Participants were classified as physically active if they reported at least 150 min/week of moderate activity, 75 min/week of vigorous activity, or an equivalent combination. Physical activity was associated with lower liver mortality among non-heavy drinkers (aSHR 0.52, 95% CI 0.28–0.94), heavy drinkers (aSHR 0.64, 95% CI 0.35–0.99), and binge drinkers (aSHR 0.31, 95% CI 0.10–0.88). The association between healthy behaviors and survival was stronger in women than in men [50]. These findings support risk modification but remain observational.

In NHANES 2017–2020, adults with hepatic steatosis were classified as MASLD *(n* = 2236), MetALD (*n* = 1355), or ALD (*n* = 457) using the SLD alcohol-exposure framework [51]. Physical activity was measured in MET-minutes per week, with high activity defined as at least 600 MET-min/week. At-risk advanced fibrosis was defined as an Agile 3+ score ≥0.45. Leisure-time, but not work-related, physical activity was associated with lower odds of at-risk advanced fibrosis in MASLD (OR 0.56, 95% CI 0.34–0.93) and combined MetALD/ALD (OR 0.55, 95% CI 0.34–0.89) [51]. These findings support the clinical relevance of leisure-time activity but do not establish an intervention effect.

Broader cohorts provide indirect support. In two prospective US cohorts of 77,238 women and 48,026 men, liver-related mortality decreased across increasing physical activity quintiles. The highest quintile had an adjusted hazard ratio of 0.46 (95% CI 0.31–0.66) relative to the lowest quintile [52]. In an accelerometer-based community cohort, each additional 30 min/day of MVPA was associated with lower odds of liver fibrosis (OR 0.57, 95% CI 0.41–0.79). The association persisted after adjustment for BMI or steatosis [53]. These studies provide indirect liver-disease evidence, not direct evidence of exercise efficacy in ALD.

## 7. Preclinical Evidence and Exercise Mechanism

Direct preclinical evidence remains limited. In mice with acute alcohol-associated liver injury, aerobic exercise reduced serum triglycerides, the liver-to-body-weight ratio, hepatic malondialdehyde, and hepatic triglyceride content. It also increased superoxide dismutase activity [8]. Treadmill exercise attenuated liver injury induced by ethanol combined with LPS and carbon tetrachloride [54]. Older rat studies also suggest that chronic ethanol ingestion and exercise training interact to affect hepatic and plasma antioxidant systems [55]. These findings support redox and inflammatory plausibility. However, these models do not reproduce chronic human ALD with AUD, malnutrition, fibrosis, sarcopenia, and social barriers.

A recent mechanistic study showed that acute treadmill exercise activated hepatic Nrf2 signaling through ROS-, AMPK-, and epinephrine-dependent pathways. Exercise also protected against acute injury induced by alcohol, acetaminophen, and carbon tetrachloride [56]. In Nrf2-deficient mice, this protection was lost, and exercise could exacerbate injury. This finding suggests that an intact adaptive stress response is necessary. The result may be clinically relevant because advanced ALD often involves reduced antioxidant reserve, malnutrition, systemic inflammation, and limited physiological tolerance.

Broader exercise biology offers additional mechanistic hypotheses. Exercise can activate AMPK- and PGC-1α-related mitochondrial remodeling, improve insulin sensitivity, and induce adaptive ROS signaling [9,10,11,12,13]. Endurance training also produces widespread multi-omic remodeling across tissues [9]. However, these observations derive mainly from healthy or non-ALD models. They should define testable mechanisms for ALD trials rather than be presented as mechanisms already demonstrated in human ALD.

## 8. Muscle–Liver Crosstalk and Sarcopenia

Skeletal muscle functions as an endocrine and metabolic organ, not merely as a target of rehabilitation [16]. In ALD, alcohol exposure, malnutrition, inflammation, inactivity, and cirrhosis converge to promote sarcopenia, impaired ammonia handling, fatigue, frailty, and adverse outcomes [18,19,57,58,59]. Muscle–liver crosstalk is therefore clinically and metabolically relevant, although the supporting evidence requires cautious interpretation.

In 301 patients across the ALD spectrum, higher myostatin and lower decorin concentrations were associated with cirrhosis, acute-on-chronic liver failure, systemic inflammation, malnutrition, reduced handgrip strength, and 30-day mortality [58]. These findings establish prognostic associations, not responsiveness to exercise. Whether exercise can modify myostatin, decorin, or related myokine profiles sufficiently to improve clinical outcomes in ALD remains unknown.

Myostatin may affect the liver by regulating the fibrogenic phenotype of hepatic stellate cells through c-Jun N-terminal kinase activation [60]. The decorin-TGF-β axis may counter profibrotic signaling [61]. These pathways suggest potential direct effects on HSCs and fibrogenesis. Skeletal muscle may also influence hepatocyte metabolism through glucose disposal, fatty acid oxidation, and lactate/alanine cycling. Effects on Kupffer cells and inflammatory signaling may occur indirectly through changes in adiposity, endotoxemia, systemic cytokines, and exercise-induced myokines. Most of these pathways remain extrapolated.

**Figure 2:**
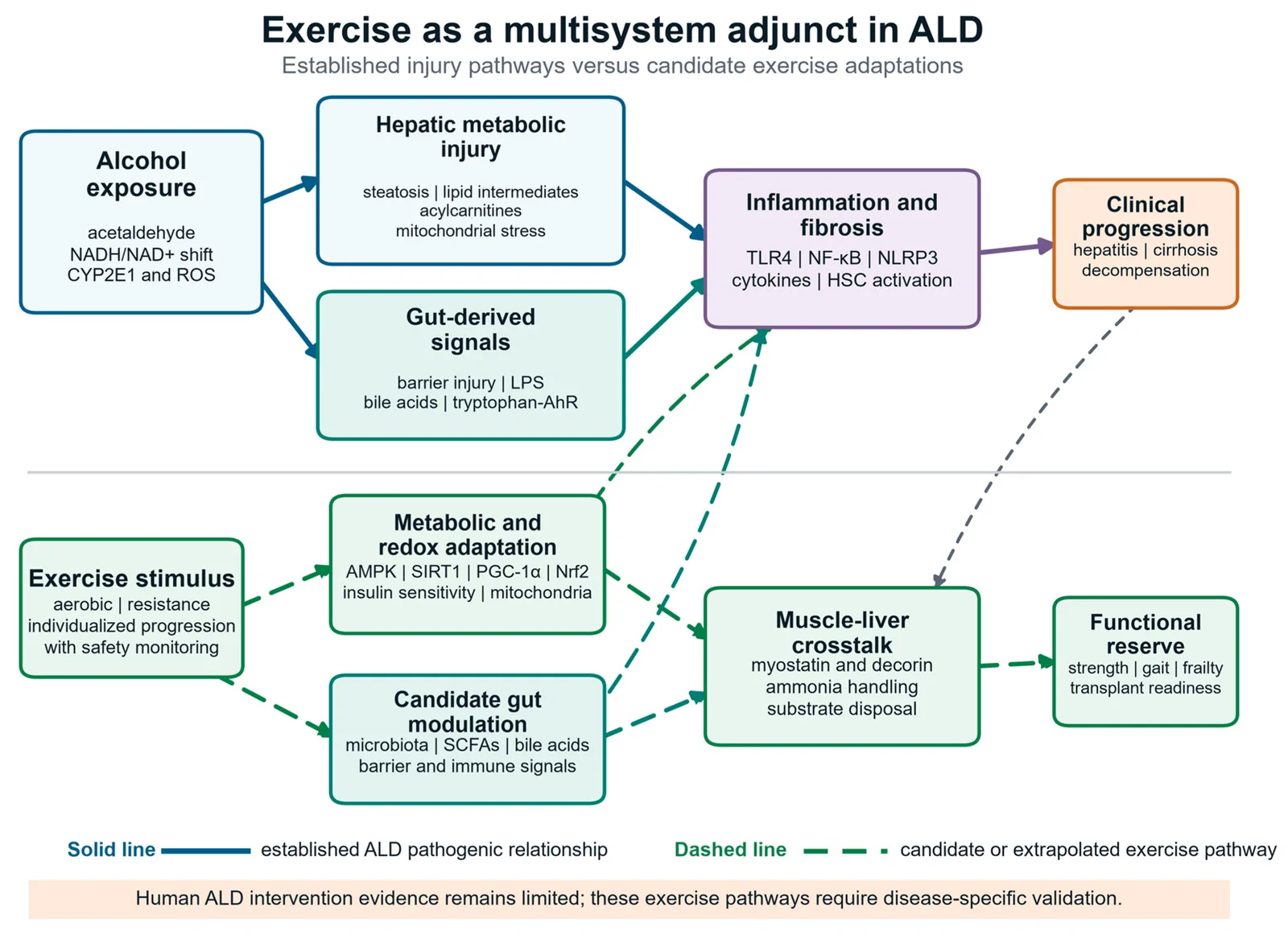
Solid arrows show established or well-supported pathogenic relationships in ALD. Dashed green and teal arrows show candidate or extrapolated exercise pathways that require ALD-specific validation. The dashed gray arrow shows the reverse influence of advanced liver disease on skeletal muscle.

Figure 2 shows exercise as a multisystem adjunct in alcohol-associated liver disease (ALD): established injury pathways and candidate adaptations.

Communication between the liver and skeletal muscle is bidirectional. Advanced liver disease can impair skeletal muscle through hyperammonemia, inflammation, reduced nutrient intake, hormonal disturbance, and inactivity [18,19,59]. Muscle loss then reduces ammonia detoxification and functional reserve, potentially increasing vulnerability to encephalopathy and hospitalization [18,19,59]. In advanced ALD, exercise may therefore improve muscle function and resilience without directly reversing hepatic injury. Figure 2 integrates the established injury pathways and candidate multisystem adaptations to exercise.

## 9. Exercise Interventions and Clinical Considerations

Translating exercise into ALD care requires safety to remain the first priority, while abstinence and AUD treatment remain central [1,2,3]. Before prescribing exercise, clinicians should assess recent alcohol use, withdrawal risk, encephalopathy, ascites, variceal bleeding risk, infection, anemia, cardiopulmonary disease, peripheral neuropathy, nutritional status, fall risk, and frailty [18,19]. Exercise should be deferred or medically supervised during unstable decompensation, active infection, severe encephalopathy, high-risk withdrawal, uncontrolled bleeding risk, or severe malnutrition.

For stable early ALD or MetALD, gradual progression toward general public health targets may be reasonable when individualized and tolerated. These targets include 150 min/week of moderate activity plus resistance training. However, they derive mainly from general physical activity and MASLD guidance rather than ALD-specific intervention trials [20,40]. The 2024 EASL-EASD-EASO MASLD guideline recommends physical activity tailored to individual capacity. It also notes that evidence for histological and liver-related clinical outcomes remains limited [20]. These targets should therefore guide supportive care and trial design rather than be presented as proven ALD therapy.

Evidence in cirrhosis is also indirect because most exercise trials enrolled mixed-etiology populations. Alcohol-specific subgroup effects and active AUD status were often not reported consistently [62,63,64,65,66,67,68,69,70]. Eight weeks of exercise training improved aerobic capacity, muscle mass, and fatigue in patients with cirrhosis [62]. Subsequent trials and reviews suggest benefits for exercise capacity, body composition, frailty, fatigue, and quality of life in selected patients [65,66,67,68,69,70]. In the Román et al. trial, home exercise was combined with BCAAs and a multistrain probiotic. Improvements in frailty therefore cannot be attributed to exercise alone [68].

Supplementary Table S2 therefore serves as a stage-specific research and practice framework, not as an evidence-based clinical recommendation for ALD. Established ALD care remains centered on abstinence, AUD treatment, nutrition, and management of complications. Exercise should be integrated as a supervised or structured adjunct with explicit monitoring. Figure 2 distinguishes established ALD pathogenic relationships from proposed and extrapolated exercise pathways by using solid and dashed arrows.

## 10. Research Gaps and Future Directions

Future research should move beyond broad lifestyle associations toward ALD-specific exercise trials. Priority populations include alcohol-associated steatosis, alcohol-associated steatohepatitis, recovery after alcohol-associated hepatitis, compensated cirrhosis, post-decompensation rehabilitation, and transplant candidates. Trials should report active alcohol use, AUD treatment status, nutrition, sex, metabolic risk, and ALDH2 genotype when relevant. They should also specify whether participants meet MASLD, MetALD, or ALD criteria [4,20,49].

Future trials should not rely on aminotransferases alone. Liver endpoints should include MRI-PDFF or CAP for steatosis, vibration-controlled transient elastography or MR elastography for stiffness, MELD score, decompensation, hospitalization, transplant-free survival, and liver-related mortality. *Metabolites*-relevant endpoints should include acylcarnitines, bile acid species, amino acid and tryptophan metabolites, lactate/pyruvate-related redox markers, lipid intermediates, short-chain fatty acids, and targeted or untargeted metabolomics [27,28,29,47]. Mechanistic and functional outcomes should include inflammatory cytokines, endotoxin and oxidative stress markers, NLRP3-related measures, myostatin, decorin, handgrip strength, gait speed, chair stands, 6 min walk distance, cardiorespiratory fitness, and adverse events.

**Figure 3:**
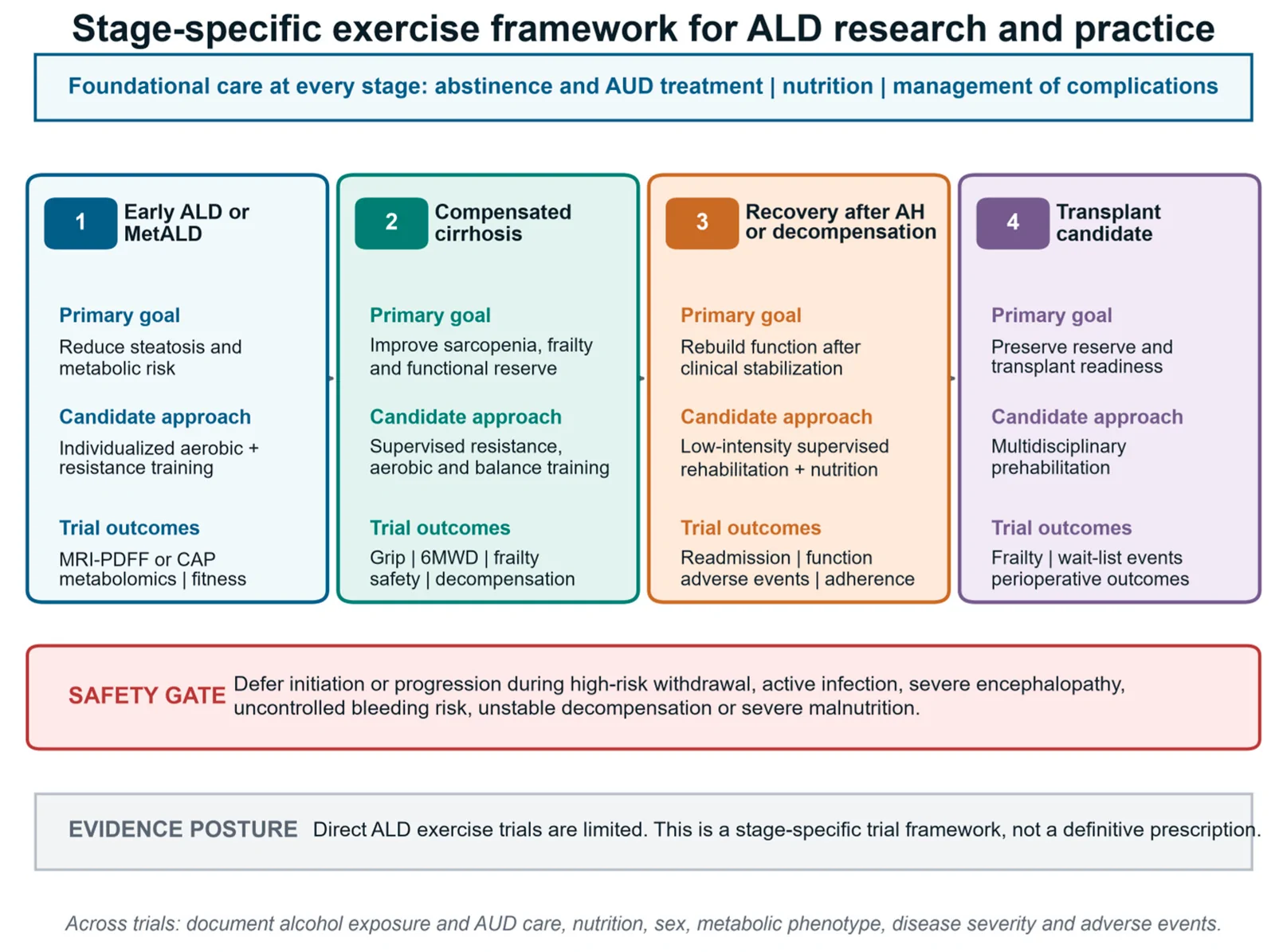
The figure aligns clinical priorities, candidate exercise approaches, and trial outcomes across early ALD or MetALD, compensated cirrhosis, recovery after alcohol-associated hepatitis or decompensation, and transplant candidacy. Foundational care and safety screening apply at every stage. Because direct ALD exercise trials remain limited, this framework is intended to guide individualized rehabilitation and study design, not to provide a definitive prescription.

Study design should reflect disease stage. Trials in early ALD and MetALD can evaluate aerobic and resistance training effects on steatosis, insulin resistance, inflammatory biomarkers, and metabolomic profiles. Trials in compensated cirrhosis should emphasize supervised progression, resistance training, balance, sarcopenia, and safety. Studies after decompensation or before transplantation should be framed as rehabilitation or prehabilitation, with nutritional support and careful adverse-event monitoring. Digital tools may support adherence monitoring and step counting, but they cannot replace clinical assessment in frail or unstable patients. Figure 3 summarizes the stage-specific priorities, safety gates, candidate approaches, and outcome domains.

The central unresolved question is whether exercise produces ALD-specific biological effects beyond its general health benefits. Answering this question requires trials that distinguish leisure-time physical activity from structured exercise training. Studies must also avoid attributing combined nutrition–probiotic–exercise effects to exercise alone and should explicitly separate direct evidence from extrapolated pathways.

Figure 3 shows a stage-specific exercise framework for alcohol-associated liver disease (ALD) research and practice.

## 11. Conclusions

Exercise remains a promising but unproven adjunctive strategy for ALD. The strongest human evidence is observational. Leisure-time physical activity is associated with lower liver mortality across drinking patterns and lower odds of at-risk advanced fibrosis in MetALD/ALD [50,51]. Direct preclinical studies support redox- and Nrf2-mediated hepatoprotection [8,54,55,56]. Mechanisms involving AMPK-SIRT1-PGC-1α, Nrf2, gut microbial metabolites, myokines, ammonia handling, and mitochondrial remodeling remain plausible but are largely extrapolated from non-ALD literature.

The evidence therefore supports a critical, metabolite-centered, and stage-aware framework rather than a definitive treatment narrative. Exercise should complement abstinence support, AUD treatment, nutrition, and safety screening. ALD-specific trials must determine whether structured training alters liver injury, metabolomic pathways, inflammation, muscle–liver signaling, physical function, and clinical outcomes.

## Data Availability

No new data were created or analyzed in this study. Data sharing is not applicable to this article.

## Supplementary Materials

The following supporting information can be downloaded at: https://www.mdpi.com/article/10.3390/metabo16090666/s1, Table S1: Evidence hierarchy for physical activity and exercise in ALD-relevant liver disease; Table S2: Stage-specific ALD exercise research and practice framework.

## Abbreviations

The following abbreviations are used in this manuscript:

**Abbreviation**	**Full term**
6MWD	6-minute walk distance
AH	alcohol-associated hepatitis
AhR	aryl hydrocarbon receptor
AI	artificial intelligence
ALD	alcohol-associated liver disease
ALDH2	aldehyde dehydrogenase 2
ALT	alanine aminotransferase
AMPK	adenosine monophosphate-activated protein kinase
AST	aspartate aminotransferase
AUD	alcohol use disorder
aHR	adjusted hazard ratio
aSHR	adjusted subdistribution hazard ratio
BCAA/BCAAs	branched-chain amino acid/amino acids
BMI	body mass index
CAP	controlled attenuation parameter
CI	confidence interval
CYP2E1	cytochrome P450 2E1
EASL-EASD-EASO	European Association for the Study of the Liver–European Association for the Study of Diabetes–European Association for the Study of Obesity
HCC	hepatocellular carcinoma
HDL	high-density lipoprotein
HE	hepatic encephalopathy
HSC/HSCs	hepatic stellate cell/cells
IL/IL-1	interleukin/interleukin-1
LPS	lipopolysaccharide
LTPA	leisure-time physical activity
MASLD	metabolic dysfunction-associated steatotic liver disease
MEDLINE	Medical Literature Analysis and Retrieval System Online
MELD	model for end-stage liver disease
MET	metabolic equivalent
MET-hours/week	metabolic equivalent-hours per week
MET-min/week	metabolic equivalent-minutes per week
MetALD	metabolic dysfunction and alcohol-associated liver disease
MR	magnetic resonance
MRI-PDFF	magnetic resonance imaging–proton density fat fraction
MVPA	moderate-to-vigorous physical activity
NADH/NAD+	reduced/oxidized nicotinamide adenine dinucleotide
NAFLD	non-alcoholic fatty liver disease
NF-κB	nuclear factor-kappa B
NHANES	National Health and Nutrition Examination Survey
NLRP3	NOD-like receptor family pyrin domain containing 3
Nrf2	nuclear factor erythroid 2-related factor 2
OR	odds ratio
PA	physical activity
PGC-1α	peroxisome proliferator-activated receptor-gamma coactivator-1 alpha
PRISMA	Preferred Reporting Items for Systematic Reviews and Meta-Analyses
RCT/RCTs	randomized controlled trial/trials
ROS	reactive oxygen species
SCFA/SCFAs	short-chain fatty acid/fatty acids
SIRT1	sirtuin 1
SLD	steatotic liver disease
TGF-β	transforming growth factor-beta
TLR4	Toll-like receptor 4
TNF-α	tumor necrosis factor-alpha
US	United States
WPA	work-related physical activity
