# Supplementary material for: Exercise as a Multisystem Adjunct for Alcohol-Associated Liver Disease: Inflammatory Mechanisms, Muscle–Liver Crosstalk, and Translational Gaps
Source: Metabolites. 2026 Sep 10;16(9):666. doi: 10.3390/metabo16090666 (PMC13609272; doi:10.3390/metabo16090666)
Supplement: Supplementary file 1 [file metabolites-16-00666-s001.zip › metabolites-4469732-supplementary.pdf]

Table S1. Evidence hierarchy for physical activity and exercise in ALD-relevant liver disease.

Category	Population/design	PA or exercise definition	Main findings	Evidence interpretation
Leisure-time PA in alcohol-exposed populations	NHANES 1984-2018; 60,334 adults; 252 liver-specific deaths; observational mortality linkage [32]	Self-reported PA; active defined as ≥150 min/week moderate, ≥75 min/week vigorous, or equivalent	Lower liver mortality in non-heavy drinkers (aSHR 0.52, 95% CI 0.28-0.94), heavy drinkers (0.64, 0.35-0.99), and binge drinkers (0.31, 0.10-0.88)	Direct human ALD-relevant association; not proof of exercise-training efficacy
Leisure-time vs work-related PA in SLD	NHANES 2017-2020; MASLD n=2236, MetALD n=1355, ALD n=457 [33]	MET-min/week; high activity ≥600 MET-min/week; LTPA analyzed separately from WPA	LTPA associated with lower at-risk advanced fibrosis in MASLD (OR 0.56, 95% CI 0.34-0.93) and MetALD/ALD (OR 0.55, 0.34-0.89); WPA not protective	Direct observational MetALD/ALD evidence; fibrosis defined by Agile 3+ ≥0.45
General liver mortality cohorts	Two prospective US cohorts; 77,238 women and 48,026 men; 295 liver-related deaths [34]	Validated weekly MET-hours of PA	Highest PA quintile aHR 0.46 (95% CI 0.31-0.66) vs lowest quintile for liver-related mortality	Indirect liver-disease evidence; not ALD-specific
Accelerometer-measured PA and fibrosis	Community cohort; n=2201; cross-sectional elastography analysis [35]	Accelerometer-derived MVPA	Each additional 30 min/day MVPA associated with lower fibrosis odds (OR 0.57, 95% CI 0.41-0.79); association persisted after BMI/steatosis adjustment	Indirect fibrosis evidence; not ALD-specific
Direct alcohol-injury exercise models	Mouse/rat alcohol-injury studies [36-39]	Aerobic or treadmill exercise; acute or chronic alcohol-related injury models	Reduced lipid accumulation/oxidative stress and Nrf2-dependent hepatoprotection in selected models	Direct preclinical evidence; limited translation to chronic human ALD

MASLD exercise trials and reviews	MASLD/NAFLD RCTs, meta-analyses, and position statement [63-70]	Structured aerobic and/or resistance training	Exercise can reduce liver fat and improve metabolic outcomes in MASLD/NAFLD	Indirect metabolic evidence; hypothesis-generating for MetALD/ALD
Mixed-etiology cirrhosis rehabilitation	Cirrhosis trials and reviews with heterogeneous etiologies [54-62]	Supervised or home-based aerobic/resistance/combined interventions	Potential improvements in aerobic capacity, muscle mass, frailty, fatigue, body composition, and quality of life	Indirect advanced-liver-disease evidence; alcohol etiology and active AUD often inconsistently reported

Abbreviations: aHR, adjusted hazard ratio; aSHR, adjusted subdistribution hazard ratio; ALD, alcohol-associated liver disease; CI, confidence interval; LTPA, leisure-time physical activity; MASLD, metabolic dysfunction-associated steatotic liver disease; MetALD, metabolic dysfunction and alcohol-associated liver disease; MET, metabolic equivalent; MVPA, moderate-to-vigorous physical activity; OR, odds ratio; PA, physical activity; SLD, steatotic liver disease; WPA, work-related physical activity.

Table S2. Stage-specific ALD exercise research and practice framework.

Stage or phenotype	Clinical definition	Evidence status	Research/practice framework	Monitoring and safety
Alcohol-associated steatosis or early MetALD	Steatosis with harmful alcohol exposure; MetALD requires metabolic dysfunction plus intermediate alcohol intake [4,64]	Mostly indirect MASLD/MetALD evidence; limited ALD trials	Reduce sedentary time; individualized aerobic plus resistance exercise as adjunct to abstinence/AUD treatment and nutrition [63-70]	Alcohol intake, AUD care, body weight, metabolic risk, CAP/MRI-PDFF, symptoms, adherence
Alcohol-associated steatohepatitis or recovery after alcohol-associated hepatitis	Inflammatory liver injury after alcohol exposure; severe hepatitis may involve jaundice and high short-term mortality [1-3,7]	No established ALD exercise prescription	Avoid unsupervised training during unstable illness; consider low-intensity rehabilitation only after clinical stabilization	MELD, bilirubin, infection, anemia, nutrition, withdrawal risk, fatigue, adverse events
Compensated cirrhosis	Cirrhosis without current ascites, bleeding, encephalopathy, or other decompensation	Indirect mixed-cirrhosis rehabilitation evidence [54-62]	Supervised or structured home-based aerobic, resistance, and balance training; prioritize sarcopenia and functional reserve	Falls, ascites/edema, perceived exertion, handgrip, chair stands, 6-minute walk distance
Decompensated cirrhosis or severe frailty	Ascites, encephalopathy, variceal bleeding, infection, renal dysfunction, or severe functional limitation	Very limited evidence; high safety heterogeneity	Medical stabilization first; low-intensity functional rehabilitation integrated with protein and micronutrient support [52,53]	Encephalopathy, infection, sodium/renal function, sarcopenia, nutrition, falls, hospitalization
Transplant candidate	Advanced ALD under transplant evaluation or waiting-list care	Prehabilitation rationale; mixed-liver-disease evidence [55,56]	Multidisciplinary prehabilitation with hepatology, nutrition, addiction medicine, and rehabilitation teams	Wait-list status, abstinence/AUD treatment, 6-minute walk distance, frailty, adverse events, post-transplant readiness

This table is a research and practice framework, not a definitive evidence-based ALD exercise prescription. Abbreviations: AUD, alcohol use disorder; CAP, controlled attenuation parameter; MELD, model for end-stage liver disease; MRI-PDFF, magnetic resonance imaging-proton density fat fraction.
